# Identification of Fibrin Clot-Bound Plasma Proteins

**DOI:** 10.1371/journal.pone.0041966

**Published:** 2012-08-03

**Authors:** Simone Talens, Frank W. G. Leebeek, Jeroen A. A. Demmers, Dingeman C. Rijken

**Affiliations:** 1 Department of Hematology, Erasmus University Medical Center, Rotterdam, The Netherlands; 2 Proteomics Center, Erasmus University Medical Center, Rotterdam, The Netherlands; Leiden University Medical Center, Netherlands

## Abstract

Several proteins are known to bind to a fibrin network and to change clot properties or function. In this study we aimed to get an overview of fibrin clot-bound plasma proteins. A plasma clot was formed by adding thrombin, CaCl_2_ and aprotinin to citrated platelet-poor plasma and unbound proteins were washed away with Tris-buffered saline. Non-covalently bound proteins were extracted, separated with 2D gel electrophoresis and visualized with Sypro Ruby. Excised protein spots were analyzed with mass spectrometry. The identity of the proteins was verified by checking the mass of the protein, and, if necessary, by Western blot analysis. Next to established fibrin-binding proteins we identified several novel fibrin clot-bound plasma proteins, including α_2_-macroglobulin, carboxypeptidase N, α_1_-antitrypsin, haptoglobin, serum amyloid P, and the apolipoproteins A-I, E, J, and A-IV. The latter six proteins are associated with high-density lipoprotein particles. In addition we showed that high-density lipoprotein associated proteins were also present in fibrinogen preparations purified from plasma. Most plasma proteins in a fibrin clot can be classified into three groups according to either blood coagulation, protease inhibition or high-density lipoprotein metabolism. The presence of high-density lipoprotein in clots might point to a role in hemostasis.

## Introduction

Arterial and venous thrombosis are major causes of morbidity and mortality in the Western world. These thrombotic disorders are considered as separate diseases, with different pathology, pathophysiology, epidemiology and treatments. However, there is evidence that suggests that there is an association between venous and arterial thrombosis [1]. Multiple genetic and acquired risk factors contribute to the development of thrombosis. Some risk factors for arterial thrombosis may also play a role in venous thrombosis and the other way around [2], [3]. Although there are several risk factors known, there are still a number of patients without a known thrombotic risk factor [4], [5]. Moreover, the site specificity of thrombosis is poorly understood [6]. Identification of novel players in hemostasis can help in determining new risk factors and additionally in understanding the pathogenesis of thrombotic disorders.

Elevated fibrinogen is a risk factor for both arterial and venous thrombosis [4], [7]. Several proteins are known to bind to fibrin and to change clot properties or clot function via effects on fibrin formation and degradation [8]. For example, the main enzyme in fibrinolysis, plasmin, is formed by activation of the zymogen plasminogen by tissue plasminogen activator (t-PA). The interactions of t-PA and plasminogen with fibrin accelerate plasminogen activation [9]. Lipoprotein(a) (Lp(a)) has structural similarities to plasminogen. Lp(a) can compete with plasminogen for fibrin binding and in so doing inhibit plasmin formation and eventually fibrinolysis [10]. Binding of proteins to fibrin can have an effect on the structure of fibrin fibers. Binding of fibronectin to fibrin causes the fibrin network to have thicker fibers and larger pores [11]. Clots with thicker fibers and larger pores are broken down more rapidly than clots with thinner fibers and smaller pores [12]. The binding of thrombin to fibrin results in a smaller quantity of active thrombin in the circulation. Reduced binding of thrombin to fibrin, which is seen in patients with fibrinogen Naples I, is associated with thrombosis [13]. There are several other proteins that are known to bind to fibrin including α_2_-antiplasmin, plasminogen activator inhibitor-2 (PAI-2), hepatocyte-derived fibrinogen-related protein-1 (HFREP-1), albumin, fibroblast growth factor-2, vascular endothelial growth factor, interleukin-1β, activated factor X, tissue factor pathway inhibitor, thrombin-activatable fibrinolysis inhibitor (TAFI), von Willebrand factor, thrombospondin, actin, factor V and factor XIII (FXIII) [14]–[21]. Some of these proteins are cross-linked to fibrin by FXIIIa, e.g. α_2_-antiplasmin, fibronectin, PAI-2, TAFI, von Willebrand factor, thrombospondin, actin and factor V.

In this study we aim to establish the protein composition of fibrin clots made from plasma. Changes in the protein composition can influence clot formation and breakdown and may therefore play a role in arterial and venous thrombosis. We identified 18 fibrin clot-bound plasma proteins by 2D gel electrophoresis followed by mass spectrometry. Nine of them were novel plasma clot components of which six proteins are associated with high-density lipoprotein (HDL).

## Materials and Methods

### Materials

Urea, thiourea, CHAPS, dithiothreitol (DTT) and iodoacetamide were obtained from Fluka (St. Louis, MO, USA). Aprotinin (Trasylol) was obtained from Bayer (Leverkusen, Germany). Tris (PlusOne), DeStreak, IPG buffer pH 3–10, immobiline strips, Ettan Spot Picker, IPGphore and Typhoon Trio apparatus were obtained from GE Healthcare (Uppsala, Sweden). The anchorchip plate, α-cyano-4-hydroxycinnamic acid matrix and the Ultraflex-II apparatus were from Bruker Daltonics (Bremen, Germany). Human thrombin and apolipoprotein A-I purified from plasma were obtained from Sigma-Aldrich (St. Louis, MO, USA). Trypsin Gold was obtained from Promega Corporation (Madison, WI, USA). Bis-Tris (12%) Criterion XT precast gels, XT MOPS and XT MES buffer were from Bio-Rad (Hercules, CA, USA). Sypro Ruby was obtained from Invitrogen (Paisley, UK) and 0.45 µm nitrocellulose transfer membrane from Whatman (Dassel, Germany). The goat polyclonal IgG to human α_2_-macroglobulin, the goat polyclonal IgG to human apolipoprotein A-II and the goat polyclonal IgG to human apolipoprotein B were from Abcam (Cambridge, UK). The rabbit polyclonal IgG to human apolipoprotein A-I was from Calbiochem (Darmstadt, Germany) and the goat polyclonal IgG to human apolipoprotein J was from Abgent (San Diego, CA, USA). The Odyssey apparatus and IRDye® 800 CW secondary donkey-anti-goat and goat-anti-rabbit antibodies were obtained from Li-Cor Bioscience (Lincoln, NE, USA). Human fibrinogen (plasminogen, von Willebrand factor and fibronectin depleted) was obtained from Enzyme Research Laboratories (South Bend, IN, USA).

### Plasma Clot Preparation

*In vitro* clots of 500 µl citrated platelet-poor plasma (pool from 10 healthy volunteers, Sanquin, location Leiden, the Netherlands) were prepared by adding calcium chloride (20 mM), thrombin (1 NIH U/ml) and aprotinin (100 KIU/ml) [15]. After 2 hours of incubation at room temperature, the clots were extensively washed by perfusing them with 10 ml Tris-buffered saline (50 mM Tris-HCl, 100 mM NaCl, pH 7.4) containing aprotinin (100 KIU/ml) at 4°C. Where indicated, the NaCl concentration was increased to 0.5 M. The clots were compacted by centrifugation, washed with deionized water and non-covalently clot-bound proteins were extracted with 150 µl rehydration buffer (7 M urea, 2 M thiourea, 4% (w/v) CHAPS, 0.5% (v/v) IPG 3–10 buffer) for 1 hour at room temperature. For optimal 2D gel electrophoresis 1% (v/v) DeStreak was added to the extract.

### 2D Gel Electrophoresis

Plasma clot extract was separated with 2D gel electrophoresis. The proteins in the 150 µl extract were separated in the first dimension with a 11 cm immobiline drystrip with a 3–10 NL pH range by isoelectric focusing on the IPGphor with the following running protocol: 30 V for 12 hours (rehydration), 1000 V for 4 hours (gradient), 8000 V for 5 hours (step-n-hold), with a 50 µA limit per gel. After isoelectric focusing the gel strip was equilibrated in buffer (6 M urea, 50 mM Tris-HCl pH 8.8, 20% (v/v) glycerol, 2% (w/v) SDS) with 1% (w/v) DTT for 15 minutes followed by a second equilibration step with equilibration buffer with 1% (w/v) iodoacetamide for 15 minutes. For the second dimension the gel strip was laid on a 12% Bis-Tris gel and run for 1 h at 200 V constant, using the XT MOPS buffer as running buffer. The proteins in the gel were visualized by Sypro Ruby staining according to manufacturer’s instructions and scanned on a Typhoon Trio at an excitation wavelength of 532 nm and an emission wavelength of 610 nm.

### Mass Spectrometry Analysis

The highly abundant proteins were analyzed with Matrix Assisted Laser Desorption/Ionization – Time of Flight (MALDI-ToF). Therefore proteins spots were excised with Spot Picker using a 2 mm picker head and destained in 30% (v/v) acetonitrile (ACN)/50 mM NH_4_HCO_3_. Destained gel pieces were vacuum-dried and rehydrated in 4 µl trypsin digest solution (75 µg/ml Trypsin Gold in 20 mM NH_4_HCO_3_, pH 8.0) for digestion overnight at room temperature. Peptide extraction was performed with 5 µl of 50% ACN/0.1% trifluoroacetic acid. The extracted sample was spotted on an anchorchip plate with saturated α-cyano-4-hydroxycinnamic acid matrix solution in 100% ACN (1∶1). Digested peptide fragments were analyzed in a MALDI-ToF mass spectrometer using an Ultraflex-II apparatus. Flexanalysis 2.4 and BioTools 3.1 software were used for data processing. The mass spectra obtained were analyzed using peptide mass fingerprint spectra with the online Matrix Science Database with MASCOT software (www.matrixscience.com). The NCBInr database 20100624 (11299630 sequences; 3855426203 residues) was searched with the Mascot parameters set as follows: Taxonomy, *homo sapiens*; mass tolerance, 100 ppm; maximally one missed cleavage per peptide; fixed modification of carboxymethylation of cysteine residues; variable modification of partial oxidation of methionine residues. Mowse scores above NCBInr database threshold of 66 were considered significant (p<0.05).

For the less abundant proteins mass spectrometry analysis was done with nanoflow LC-MS/MS. Picked gel spots were subjected to in-gel reduction with DTT, alkylation with iodoacetamide and digestion with Trypsin Gold, essentially as described by Wilm *et al.* [22]. Nanoflow LC-MS/MS was performed on an 1100 series capillary LC system (Agilent Technologies, Santa Clara, CA, USA) coupled to an LTQ linear ion trap mass spectrometer (Thermo Fisher Scientific, Waltham, MA, USA) operating in positive mode and equipped with a nanospray source. Peptide mixtures were trapped on a ReproSil C18 reversed phase column (Dr Maisch GmbH, Ammerbuch-Entringen, Germany; column dimensions 1.5 cm×100 µm, packed in-house) at a flow rate of 8 µl/min. Peptide separation was performed on ReproSil C18 reversed phase column (Dr Maisch GmbH, Ammerbuch-Entringen, Germany; column dimensions 15 cm×50 µm, packed in-house) using a linear gradient from 0 to 80% B (A = 0.1% formic acid; B = 80% (v/v) acetonitrile, 0.1% formic acid) in 70 min and at a constant flow rate of 200 nl/min using a splitter. The column eluent was directly sprayed into the ESI source of the mass spectrometer. Mass spectra were acquired in continuum mode; fragmentation of the peptides was performed in data-dependent mode. Peak lists were automatically created from raw data files using the Mascot Distiller software (version 2.1; MatrixScience). The Mascot search algorithm (version 2.2, MatrixScience) was used for searching against the NCBInr database (release NCBInr_20090808.fasta; taxonomy: *Homo sapiens*). The peptide tolerance was typically set to 2 Da and the fragment ion tolerance was set to 0.8 Da. A maximum number of 2 missed cleavages by trypsin were allowed and carbamidomethylated cysteine and oxidized methionine were set as fixed and variable modifications, respectively. The Mowse score cut-off value for a positive protein hit was set to 60. For identification as fibrin clot-binding protein the cut-off value for the emPAI score [23] was set at 0.15. Individual peptide MS/MS spectra with Mascot scores below 40 were checked manually and either interpreted as valid identifications or discarded.

### Fibrinogen Purification from Plasma

Fibrinogen was purified from barium-adsorbed citrated plasma with immunoaffinity chromatography according to Takebe et al. [24] with some changes. In short, IF-1 antibody was conjugated to CNBr-activated Sepharose 4B according to manufacturer’s manual. Barium-adsorbed citrated plasma was dialyzed against Tris-buffered saline (50 mM Tris-HCl, 100 mM NaCl, pH 7.4) and applied on the IF-1-conjugated Sepharose 4B column with 1 mM CaCl_2_. The column was washed with 50 mM Tris-HCl, pH 7.4, containing 0.3 M NaCl and 1 mM CaCl_2_ and eluted with 50 mM Tris-HCl, pH 7.4, containing 0.3 M NaCl and 5 mM EDTA. The optical densities at 280 nm of the fractions were measured.

### Western Blot Analysis

To detect α_2_-macroglobulin, a clot extract was made and separated by 2D gel electrophoresis as described above. The proteins were transferred from the 12% Bis-Tris precast gel to a nitrocellulose membrane by semi-dry blotting at 0.33 mA constant for 1 hour. The membrane was incubated with block buffer (PBS, 1% BSA, pH 7.4). After the blocking step the membrane was first incubated with the α_2_-macroglobulin antibody (20 µg/ml) diluted in block buffer containing 0.1% Tween 20 and then with the secondary antibody IRDye® 800 CW donkey-anti-goat diluted 10.000 times in 5% milk and 0.1% Tween 20 in PBS, pH 7.4. All incubation steps were performed for 1 hour at room temperature. To visualize the protein, the membrane was scanned on an Odyssey scanner.

To detect fibrinogen-bound proteins, fibrinogen in the fractions of the IF-1 column experiment described above and fibrinogen from Enzyme Research Laboratories were analyzed with SDS-PAGE. Reduced samples of 30 µg fibrinogen were run on a 12% Bis-Tris precast gel with XT MES buffer for 1 h at 200 V constant and analyzed with Western blotting as described above. The different apolipoproteins were detected by using the specific apolipoprotein A-I antibody (1000 times diluted), apolipoprotein J antibody (0.5 µg/ml) and apolipoprotein A-II antibody (1 µg/ml).

The amounts of apolipoprotein A-I and apolipoprotein B present in plasma clot extracts were estimated using Western blot analysis. For quantification of apolipoprotein A-I a clot extract and different concentrations of purified apolipoprotein A-I were run on a Tris-HCl gel (15%) and for apolipoprotein B quantification a clot extract and different concentrations of purified low-density lipoprotein (LDL) were run on a Tris-HCl gel (5%). LDL was purified according to Redgrave et al. [25]. Calibration curves were made of the different concentrations of apolipoprotein A-I and apolipoprotein B which were used to estimate the amount of apolipoprotein in the plasma clot extract. Western blot analysis was done as described above using a specific apolipoprotein A-I antibody (1000 times diluted) and a specific apolipoprotein B antibody (1 µg/ml).

## Results

**Figure 1:**
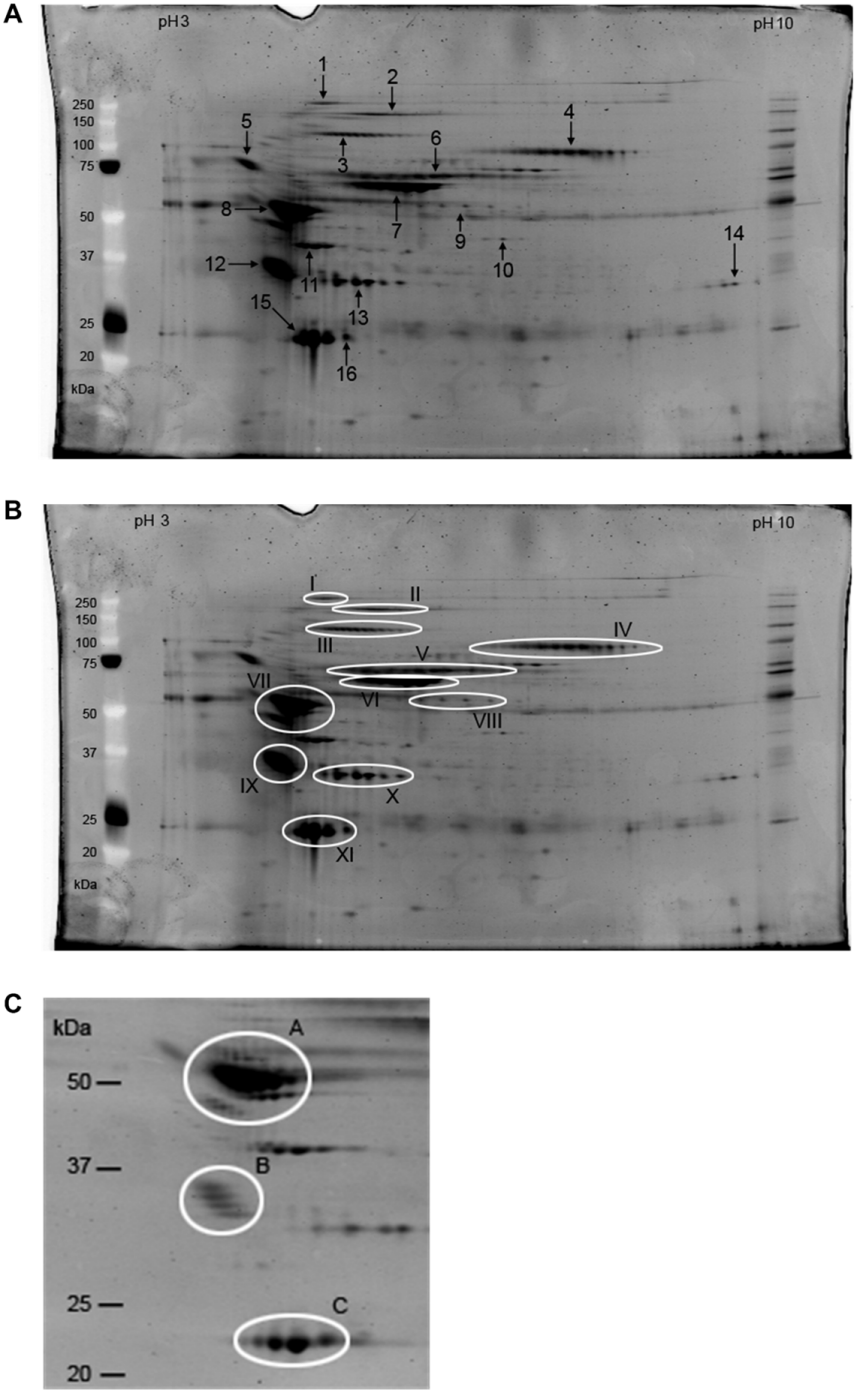
An overview of non-covalently fibrin clot-bound plasma proteins. Plasma clots were made by adding CaCl_2_, thrombin and aprotinin to platelet-poor citrated normal plasma, unbound proteins were washed away and bound proteins were extracted. These proteins were separated with 2D gel electrophoresis and visualized by Sypro Ruby. A) The numbers and arrows indicate the protein spots that were excised from gel and analyzed with mass spectrometry. B) The trains of spots that resemble the same protein are indicated by white ellipses. They include: fibronectin (I), α_2_-macroglobulin (II, III and VIII), plasminogen (IV), FXIII A chain (V), albumin (VI), α_1_-antitrypsin (VII), apolipoprotein J (IX), apolipoprotein E, HFREP-1 (X) and apolipoprotein A-I (XI). C) A zoomed image of the 2D gel with a lower fluorescent signal. The isoforms of α_1_-antitrypsin (A), apolipoprotein J (B) and apolipoprotein A-I (C) are indicated by white ellipses.

To investigate the protein composition of a fibrin clot, *in vitro* plasma clots were made by adding CaCl_2_, thrombin and aprotinin to platelet-poor citrated plasma. Unbound proteins were washed away and non-covalently bound proteins were extracted, separated with 2D gel electrophoresis and visualized with Sypro Ruby (Figure 1A). Spots that were identified using mass spectrometry were reproducibly detected in at least 7 out of 10 2D gels. The high-abundant protein spots (spots 7, 8 and 15) were analyzed with MALDI-ToF mass spectrometry and the other protein spots were analyzed with nanoflow LC-MS/MS. This resulted in the identification of 18 different proteins that were present in a plasma clot. Detailed information from Mascot analysis is shown in table 1. Several of the proteins identified were not previously described as plasma clot components including α_2_-macroglobulin, carboxypeptidase N (CPN), α_1_-antitrypsin, haptoglobin, serum amyloid P and the apolipoproteins A-I, A-IV, E and J. The latter six proteins are associated with the HDL particle. In addition we identified proteins that have previously been described as fibrin clot-bound proteins, including fibronectin, plasminogen, factor XIII, HFREP-1, actin and thrombin.

**Table 1:** Mascot analysis of fibrin clot-bound proteins.

Spot	Accession #	Description	Mowse score	Seq. cov. (%)	Mw	Tot. pept.	Uniq. pept.	pI
1	gi|16933542	Fibronectin	2025	20	262656	33	30	5.49
2	gi|46812315	α_2_-macroglobulin	2206	29	167505	43	33	6.06
3	gi|46812315	α_2_-macroglobulin	1996	24	167505	46	29	6.06
4	gi|190026	Plasminogen	2240	49	93233	39	34	7.04
5	gi|51173528	carboxypeptidase N, polypeptide 2	864	29	61433	30	13	5.72
6	gi|119395709	coagulation factor XIII, A1 polypeptide	864	21	83267	39	15	5.75
	gi|33451	immunoglobulin heavy constant mu	555	21	51506	27	9	5.92
7	gi|4502027	albumin (*)	280	54	71317	28	25	5.92
8	gi|15080499	α_1_-antitrypsin (*)	162	45	46864	15	13	5.36
9	gi|46812315	α_2_-macroglobulin	621	7	167505	10	8	6.06
	gi|113584	immunoglobulin heavy constant alpha 1	282	13	38486	5	4	6.08
10	gi|4503011	carboxypeptidase N, polypeptide 1	210	7	52538	4	3	6.86
11	gi|4501887	actin, gamma 1	693	33	42108	20	10	5.31
	gi|178759	apolipoprotein A-IV	424	14	45307	6	6	5.23
	gi|306882	haptoglobin	209	8	45860	3	3	6.24
12	gi|177827	α_1_-antitrypsin	373	12	46787	7	7	5.42
	gi|338305	apolipoprotein J	344	16	36997	7	5	5.74
13	gi|178849	apolipoprotein E	1124	53	36302	32	17	5.65
	gi|22023090	HFREP-1	448	26	36640	8	7	5.58
14	gi|38018090	thrombin	599	23	34072	11	8	8.52
15	gi|4557321	apolipoprotein A-I (*)	153	49	30759	18	15	5.56
16	gi|178775	apolipoprotein A-I	723	44	28944	15	11	5.45
	gi|149673887	immunoglobulin light chain	442	40	23665	7	5	6.97
	gi|337758	serum amyloid P	306	18	25495	4	4	6.10

Protein spots shown in Fig. 1A were analyzed by mass spectrometry. Proteins with an asterisk were analyzed with MALDI-ToF and the other protein spots were analyzed with nanoflow LC-MS/MS. Accession number of the NCBInr database, protein description, Mowse score, sequence coverage (%), calculated molecular weight (Mw), total identified peptides, unique identified peptides and the calculated pI are given.

HFREP-1; hepatocyte-derived fibrinogen related protein-1.

The majority of the identified spots belong to a train of spots, as is seen for example for protein spot 4 in figure 1A. These trains most likely represent different isoforms of the same protein. On the basis of multiple mass spectrometry analyses of different spots from different gels we identified several trains of spots (figure 1B). To visualize low-abundant fibrin clot-bound proteins on the 2D gel, the laser intensity in figure 1A was set high. The disadvantage of the resulting high fluorescent signal was that the high-abundant proteins displayed a saturated signal. Therefore we show in figure 1C a zoomed image of the high-abundant proteins with a lower fluorescent signal. Although α_1_-antitrypsin (spot A in figure 1C) mainly varied in its isoelectric point, several minor species with higher and lower molecular mass were also detected.

**Figure 2:**
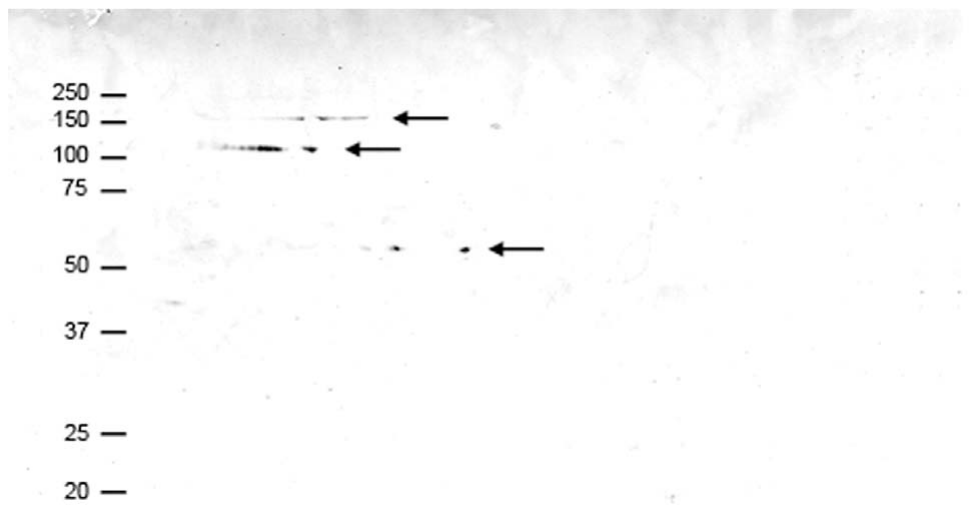
Western blot analysis with specific α_2_-macroglobulin antibodies. Fibrin clot-bound plasma proteins were separated with 2D gel electrophoresis and analyzed with Western blot analysis using specific α_2_-macroglobulin antibodies. The arrows indicate the three different α_2_-macroglobulin trains that were also identified as α_2_-macroglobulin with mass spectrometry (protein spots 2, 3 and 9 in figure 1A and table 1). The molecular mass of the protein marker is indicated in kDa.

The identification by mass spectrometry was verified by comparing the theoretical molecular mass of the proteins, obtained from the NCBInr database, with the apparent mass on the 2D gel. For most protein spots the theoretical molecular mass was similar to the mass estimated from the gel pattern. However, of three protein spots (2, 3 and 9 in figure 1A) identified as α_2_-macroglobulin only one spot (spot 2) corresponded with the theoretical mass of the protein of 167 kDa. With Western blot analysis, using α_2_-macroglobulin specific antibodies, the identity of the three protein spots and their isoforms in the same trains were confirmed to be α_2_-macroglobulin (figure 2).

**Figure 3:**
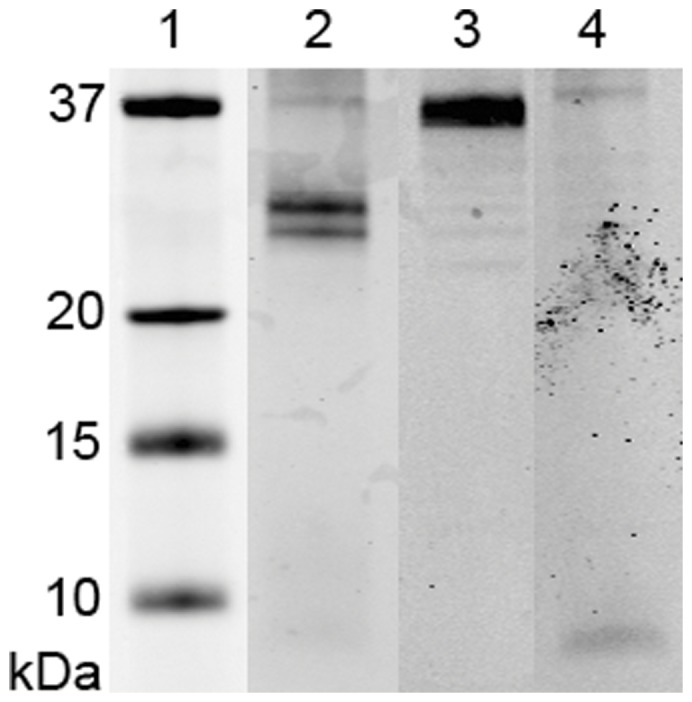
Western blot analysis for apolipoproteins in purified fibrinogen. Fibrinogen was isolated from plasma with immunoaffinity chromatography and run on SDS-PAGE. Different apolipoproteins were detected with Western blot analysis using specific antibodies. Lane 1: protein marker, lane 2: apolipoprotein A-I (Mw = 28,900), lane 3: apolipoprotein J (Mw = 37,000), lane 4: apolipoprotein A-II (Mw = 8,700). Only the relevant section of the gel is shown.

It was a remarkable observation that two-thirds of the novel plasma clot components appeared to be HDL-associated proteins. Next to the presence of these proteins in a fibrin clot we detected the presence of HDL-apolipoproteins in fibrinogen that we purified from plasma (figure 3) and in commercially available fibrinogen purified form plasma (data not shown). This suggests that HDL was bound specifically to fibrinogen and thereby to the plasma clot, which was also supported by the finding that HDL-apolipoproteins present in a plasma clot could not be washed out by increasing the NaCl in the washing buffer from 0.1 M to 0.5 M (data not shown). The amount of apolipoprotein A-I present in a washed plasma clot, estimated from Western blot analysis of clot extracts was about 3 µg/ml. This corresponded to approximately 8 µg HDL per ml plasma. The amount of apolipoprotein A-I was much higher than apolipoprotein B (about 0.05 µg/ml), which corresponded to approximately 0.3 µg LDL per ml plasma clot.

## Discussion

In this study, using 2D gel electrophoresis and mass spectrometry, we identified 18 different fibrin clot-bound proteins, which are not cross-linked to fibrin by FXIIIa. Several of these protein have not been described before as plasma clot components.

Eleven out of the 18 fibrin clot-bound proteins can be classified into three groups related to their function: blood coagulation, protease inhibition and HDL metabolism. Plasminogen, factor XIII and thrombin are involved in blood coagulation while α_2_-macroglobulin and α_1_-antitrypsin are protease inhibitors [26], [27]. Plasma proteins that are associated with HDL and play a role in its metabolism are haptoglobin, serum amyloid P and the apolipoproteins A-I, A-IV, J and E [28]–[31]. The presence of actin as a plasma clot component could be due to small amounts of platelets present in the platelet-poor plasma. However, actin can also be released into the bloodstream by dying cells or tissue damage [32].

The intensity of the stained spots suggests that the 18 identified plasma proteins represent nearly the entire protein material non-covalently bound to a fibrin clot. However with this approach we do not visualize the very low-abundant proteins. For example t-PA, a known fibrin-binding protein, was not observed. A second limitation of 2D gel electrophoresis is that high molecular weight proteins are underrepresented [33]. However, we did observe the high molecular weight proteins fibronectin and α_2_-macroglobulin with 2D gel electrophoresis. In addition, with 1D gel electrophoresis (SDS-PAGE) and protein staining we did not observe any additional high molecular weight protein in a plasma clot extract (data not shown).

To verify the identification of the protein spots by mass spectrometry the theoretical molecular mass of the identified protein was compared with the apparent molecular mass on the 2D gel. For most proteins the theoretical molecular mass was comparable with the observed molecular mass, given that the theoretical mass does not take into account several post-translational modifications like glycosylation. Three different protein spots on the 2D gel of figure 1A were identified as α_2_-macroglobulin and the molecular mass of only one protein spot was comparable with the theoretical mass of the protein. However, all protein spots identified as α_2_-macroglobulin were confirmed with Western blot analysis. It has been shown that when α_2_-macroglobulin is heated at high temperatures or at lower temperatures under denaturing conditions, two polypeptide chains of 125 kDa and 62 kDa can be produced [34]. Although we did not heat the clot extract in the preparation for 2D gel electrophoresis, the molecular weights of the two α_2_-macroglobulin fragments we observed match the molecular weights of the fragments produced upon heating.

Very recently, after we finished the research described here, a paper was published that described complement C3 as a novel plasma clot component [35]. Most proteins identified were related to coagulation and inflammation, while we identified mainly proteins that were related to coagulation, protease inhibition and HDL metabolism. There are some clear differences between the two proteomic approaches. The most important difference is that Howes et al. [35] described the total protein composition of the whole clot, thereby also identifying proteins that are crosslinked via FXIIIa, while we focused on non-covalently plasma clot-bound proteins. Identifying proteins by examining the whole clot is technically more challenging because of the high abundance of fibrin compared to the other plasma clot components.

Two-thirds of the newly identified fibrin clot-bound proteins are associated with HDL suggesting that HDL particles have affinity for fibrin, which is specific for HDL and not for LDL because only low amounts of apolipoprotein B were present in a fibrin clot. In addition, this apolipoprotein B most likely comes from bound lipoprotein(a), which can bind with its apolipoprotein(a) to fibrin [10]. The presence of HDL-proteins in purified fibrinogen suggested affinity of HDL to fibrinogen as well. These findings are in line with the detection of fibrinogen in purified HDL preparations [29], [36], [37]. What the role is of the binding of HDL to a fibrin clot is not known. However, recent studies have shown that HDL levels are negatively associated with both arterial and venous thrombosis [38]–[40], for which the exact mechanism is not known. HDL is a reverse cholesterol transporter, which is considered to be the most important property of HDL in preventing atherosclerosis. Several other properties can contribute to the atheroprotective effect of HDL including antioxidant, anti-inflammatory, antiproliferative, antithrombotic and vasodilatory properties [41]. HDL consists of a heterogeneous population of particles containing different types and amounts of (apolipo)proteins and lipids. The existence of different subpopulations in HDL is consistent with the fact that HDL has multiple biological activities [42]. It is possible that the HDL particle present on a fibrin clot as identified in this study represents a distinct subfraction of HDL.

A direct role of HDL in coagulation or fibrinolysis is not yet clear. It was suggested that HDL enhances the activated protein C pathway [43], but this may be due to the contamination of negatively charged phospholipid membranes [44]. Another possible mechanism that can play a role in the anticoagulant effect of HDL is that anionic phospholipids lose their procoagulant properties when incorporated into HDL [45].

In conclusion, we have identified several novel plasma clot components of which two-thirds was associated with HDL particles. This suggests that the presence of HDL on a fibrin clot may be of importance in clot formation or fibrinolysis and may play a role in the hemostasis and thrombosis.
